# First record of the genus *Aetius* O. Pickard-Cambridge, 1897 from China, with description of a new species (Araneae, Corinnidae, Castianeirinae)

**DOI:** 10.3897/BDJ.10.e96594

**Published:** 2022-12-06

**Authors:** Lu Zhang, Yannan Mu, Feng Zhang

**Affiliations:** 1 The Key Laboratory of Zoological Systematics and Application, Institute of Life Science and Green Development, College of Life Sciences, Hebei University, Baoding, China The Key Laboratory of Zoological Systematics and Application, Institute of Life Science and Green Development, College of Life Sciences, Hebei University Baoding China

**Keywords:** morphology, Dionycha, taxonomy, Yunnan Province

## Abstract

**Background:**

The genus *Aetius* O. Pickard-Cambridge, 1897 has been discovered in Sri lanka, India, Malaysia (Borneo), Thailand and Ivory Coast and comprises four species: *A.bicuspidatus* Yamasaki, 2020, *A.decollatus* O. Pickard-Cambridge, 1897, *A.nocturnus* Deeleman-Reinhold, 2001 and *A.tuberculatus* (Haddad, 2013).

**New information:**

The genus *Aetius* O. Pickard-Cambridge, 1897 is reported for the first time in China (Yunnan Province) and includes three species: one new species and two newly-recorded species. One new species is described, based on both sexes: *A.maculatus* **sp. n**. Additionally, *A.bicuspidatus* Yamasaki, 2020 and *A.nocturnus* Deeleman-Reinhold, 2001, are newly recorded from China, with photographs of habitus and genitalia being provided.

## Introduction

Pickard-Cambridge (1897) erected the genus *Aetius* based on the female holotype. The genus was later considered as a member of Castianeirinae Clubionidae Wagner, 1887) by Reiskind (1969). Then Deeleman-Reinhold (2001) placed it in the family Corinnidae Karsch, 1880.

O. Pickard-Cambridge described *Aetiusdecollatus* with the female specimen, but did not provide detailed description of genital characteristics (Pickard-Cambridge 1897). Subsequently, Reimoser (1934) described the male of *A.decollatus*, but neither detailed description nor illustration were provided. Majumder and Tikader (1991) checked the holotype of *A.decollatus* and provided a detailed description of the genitalia. *Sudhin et al. (2016)* described *A.decollatus* with both sexes, redescribed the female, based on fresh material and explained and illustrated a new characteristic, the semicircular cymbial notch of the male palp.

Deeleman-Reinhold (2001) described *Aetiusnocturnus* on a single female specimen from Borneo. Dankittipakul and Singtripop (2013) described the male of *A.nocturnus* for the first time. *Aetiustuberculatus* (Haddad, 2013) was originally identified as a member of *Apochinomma* (Haddad 2013), afterwards Caleb and Mathai (2016) transferred it from *Apochinomma* to *Aetius* according to somatic and genital morphology. *Yamasaki et al. (2020)* described the fourth species of this genus, *A.bicuspidatus*, from Borneo, with both sexes.

While examining corinnid specimens collected from Yunnan Province, China in recent years, we found a new species, *A.maculatus* **sp. n**., which is consistent with the generic characteristics of *Aetius*, representing this genus to be the first recorded from China. Two known species, *A.bicuspidatus* Yamasaki, 2020 and *A.nocturnus* Deeleman-Reinhold, 2001, were also recorded from China for the first time, representing the northernmost record for the genus.

## Materials and methods

All measurements are given in millimetres (mm). Leg measurements are shown as total length (femur, patella, tibia, metatarsus, tarsus). The total length provided here is an estimate, based on the sum of the carapace and abdomen, excluding the pedicel (Haddad 2013). Epigynes were removed and cleared in a pancreatin solution (Álvarez-Padilla and Hormiga 2007). All specimens were preserved in 95% alcohol and were examined, illustrated and measured with a Leica M205A stereomicroscope. Somatic photographs were captured using a Leica M205A stereomicroscope, equipped with a DFC550 CCD camera and genital morphology photographs were taken using an Olympus BX51 microscope, equipped with a Kuy Nice CCD with a Canon 60mm micro-lens and were imported into Helicon Focus v. 7 for stacking. Final figures were retouched with Adobe Photoshop CC © 2022. Distribution map was made using the ArcGIS Desktop version 10.6. The specimens used in this study are deposited in the Museum of Hebei University, Baoding, China (MHBU).

The abbreviations used in the text are as follows: Eyes: ALE = anterior lateral eye; AME = anterior median eye; MOA = median ocular area; PLE = posterior lateral eye; PME = posterior median eye.

## Taxon treatments

### Aetius

O. Pickard-Cambridge, 1897

CB942E70-0964-5F42-A4C4-8C5290C70BA4

#### Diagnosis

*Aetius* is most similar to *Serendib* Deeleman-Reinhold, 2001 in having eight eyes in two rows, posterior eye row wide and strongly recurved, but can be distinguished by:

1. carapace sub-pentagonal, widest at coxae II and III; posterior of carapace narrowed, forming trapezoidal extension over pedice;
2. legs slender, with spines; legs formula 4, 2, 1, 3;
3. abdomen oval or round, with large dorsal scutum, laterally constricted, posteriorly gradually widened; anteriorly without strong erect spines;
4. male palp with baso-retrolateral semi-circular cymbial notch.

### Aetius maculatus sp. n.

94A14EF3-64B7-5C56-8645-E0858861DFDD

D8F4CFA8-FE94-4787-BDF1-FBE6A17EFD80

#### Materials

- **Type status:** Holotype. **Occurrence:** recordedBy: Weihang Wang; individualCount: 1; sex: male; lifeStage: adult; occurrenceID: 29C90E69-17CE-594E-9AA3-3E5CDAF65D92; **Location:** country: China; stateProvince: Yunnan Province; county: Jinghong; locality: Wild Elephant Valley; verbatimElevation: 814; verbatimLatitude: 22°10′25.05″N; verbatimLongitude: 100°51′19.07″E; **Event:** year: 2022; month: 6; day: 5; **Record Level:** institutionID: the Museum of Hebei University; institutionCode: MHBU
- **Type status:** Paratype. **Occurrence:** recordedBy: Ku Yu; individualCount: 1; sex: female; lifeStage: adult; occurrenceID: 782A0534-2547-5D0F-BE56-F27DFDCCC2C8; **Location:** country: China; stateProvince: Yunnan Province; county: Jinghong; locality: Wild Elephant Valley; verbatimElevation: 800; verbatimLatitude: 22°10′25.05″N; verbatimLongitude: 100°51′19.07″E; **Event:** year: 2021; month: 8; day: 3; **Record Level:** institutionID: the Museum of Hebei University; institutionCode: MHBU

#### Description

**Figure 1.**
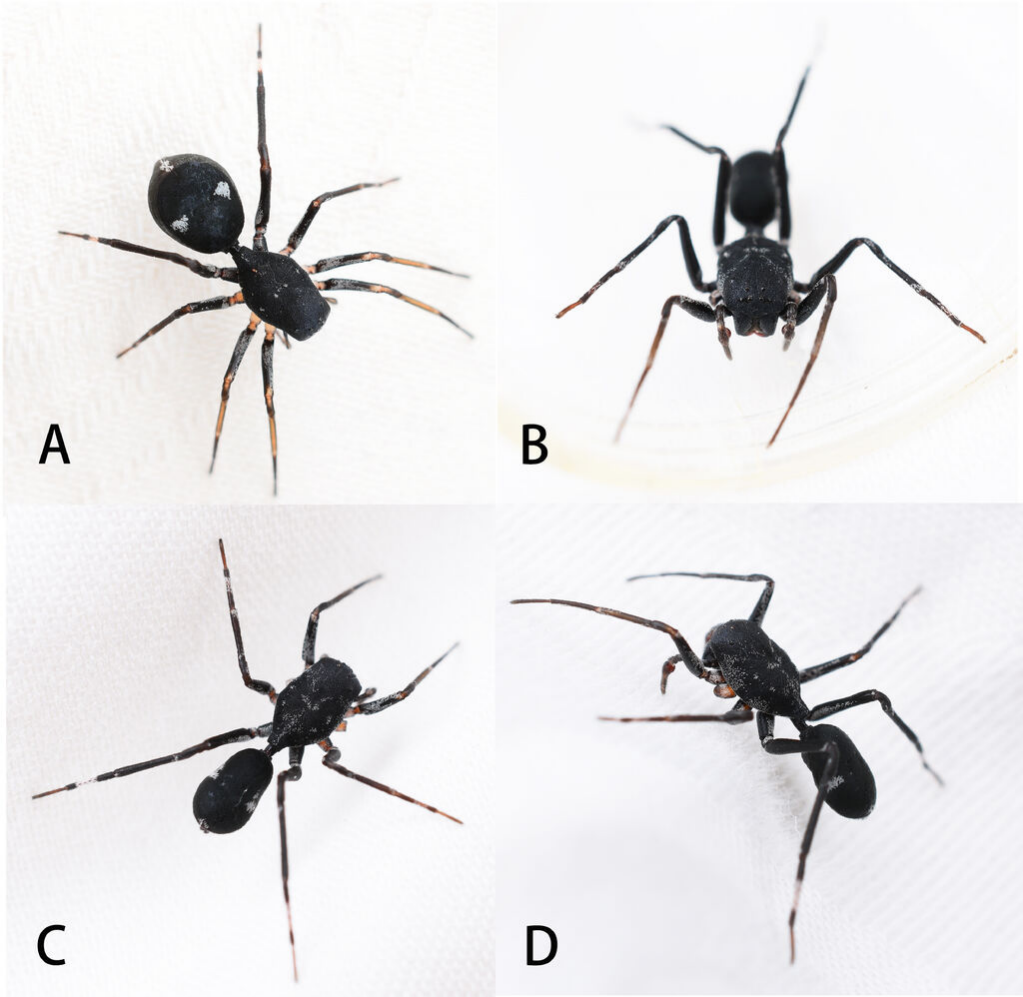
Living habitus of *Aetiusmaculatus* **sp. n. A** female; **B–D** male (photographs by Weihang Wang).

**Figure 2.**
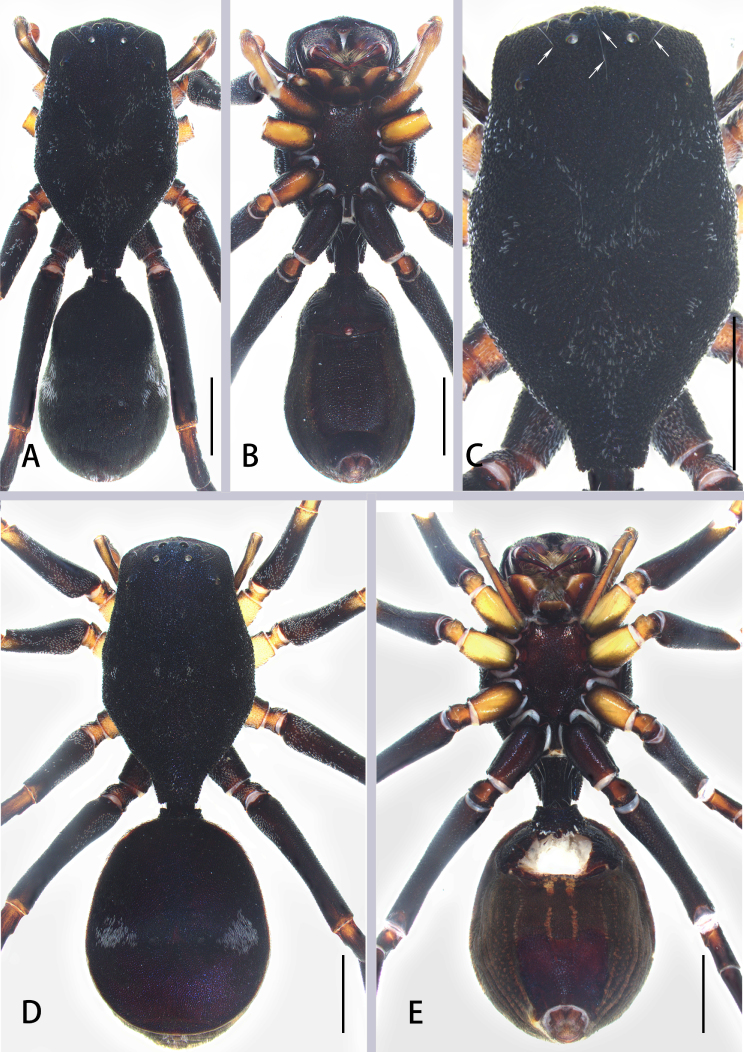
*Aetiusmaculatus* **sp. n. A** male habitus, dorsal view; **B** same, ventral view; **C** male carapace, dorsal view; **D** female habitus, dorsal view; **E** same, ventral view. Scales = 0.5 mm (A–E).

Male (Holotype) (Fig. 1B–D and Fig. 2A–C). Total length 6.91; carapace 3.72 long, 2.15 wide; abdomen 3.19 long, 1.98 wide. Eye sizes and interdistances: AME 0.16, ALE 0.11, PME 0.10, PLE 0.09; AME–AME 0.36, AME–ALE 0.21, ALE–ALE 0.72, PME–PME 0.49, PME–PLE 0.57, PLE–PLE 1.37, ALE–PLE 0.63; MOA 0.21 long; anterior width 0.48, posterior width 0.58. Clypeal height 0.43. Chelicerae with two pro- (proximal smallest, distal largest) and two retromarginal teeth (same size). Labium 0.21 long, 0.39 wide; sternum 1.32 long, 0.97 wide. Measurements of legs: I 7.14 (1.91, 0.70, 1.76, 1.43, 1.34), II lost, III 6.65 (2.01, 0.77, 1.69, 1.37, 0.81), IV 9.03 (2.69, 0.77, 2.50, 2.02, 1.05).

Carapace black, sub-pentagonal, sclerotised, widest at coxae II and III, surface with white, sparse plumose hair; ocular region with white setae; posterior of carapace narrowed, forming trapezoidal extension over pedicel (Fig. 2C). Legs dark brown, slender, all femora black, with white feathery setae dorsally, tibia Ⅰ-Ⅱ and middle of metatarsi Ⅳ yellow. Abdomen oval, laterally constricted, posteriorly gradually widened; both sides with pinnate hairs, forming two white patches and posterior with a white patch. Dorsal scutum large, nearly covered abdomen (Fig. 2A); epigastric scutum extending anteriorly and sclerotised, forming short, grooved collar ring; ventral scutum rectangular, heavily sclerotised (Fig. 2B).

**Figure 3.**
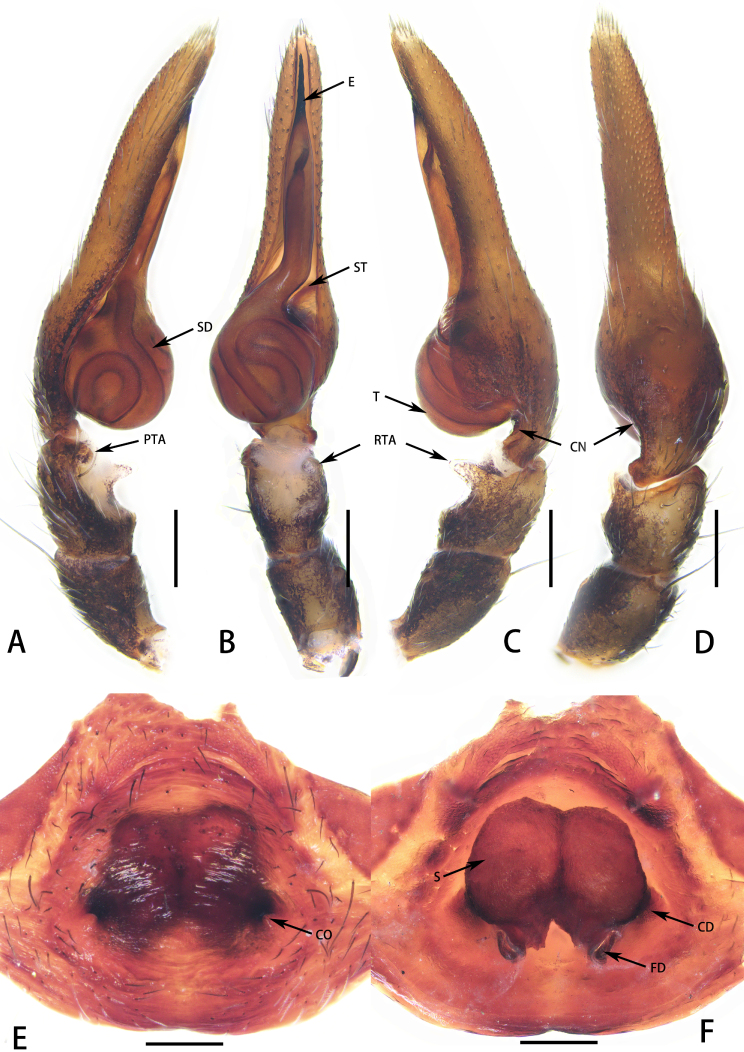
*Aetiusmaculatus* **sp. n. A** Male left palp, prolateral view; **B** Same, ventral view; **C** Same, retrolateral view; **D** Same, dorsal view; **E** epigyne, ventral view; **F** same, dorsal view. Abbreviations: CD—copulatory duct; CO—copulatory opening; CN—cymbial semi-circular notch; E—embolus; FD—fertilisation duct; PTA—prolateral tibial apophysis; RTA—retrolateral tibial apophysis; S—spermatheca; SD—sperm duct; ST—subtegulum; T—tegulum. Scales = 0.1 mm (**A–F**).

Palp (Fig. 3A–D). Tibia concaved in ventral surface; distal margin developed, forming prominence; retrolateral tibial apophysis digitiform, weakly sclerotised, slightly transparent; prolateral tibial apophysis semicircular. Embolus conical, elongate, slender distally, with screw thread surface.

Female (Fig. 2D and E). Total length 8.25; carapace 4.22 long, 2.48 wide; abdomen 4.03 long, 2.97 wide. Eye sizes and interdistances: AME 0.15, ALE 0.12, PME 0.11, PLE 0.08; AME–AME 0.37, AME–ALE 0.22, ALE–ALE 0.80, PME–PME 0.53, PME–PLE 0.61, PLE–PLE 1.48, ALE–PLE 0.63; MOA 0.24 long; anterior width 0.47, posterior width 0.60. Clypeal height 0.53. Labium 0.33 long, 0.57 wide; sternum 1.62 long, 1.22 wide. Measurements of legs: I 7.86 (2.15, 0.70, 2.12, 1.67, 1.22), II 7.91 (2.29, 0.75, 2.03, 1.61, 1.23), III 7.62 (2.34, 0.79, 2.01, 1.56, 0.92), IV 10.2 (2.99, 0.92, 2.87, 2.28, 1.14).

As in male, except abdomen nearly round, with two white spots and dorsal scutum large, bluish-violet, extending five-sixths the length of dorsum.

Epigyne (Fig. 3E and F): simple, strongly sclerotised, with copulatory opening, small, C-shaped. Copulatory duct extremely short, connecting to spermatheca. Spermatheca fabaceous, with fertilisation duct posteriorly.

#### Diagnosis

**Figure 4.**
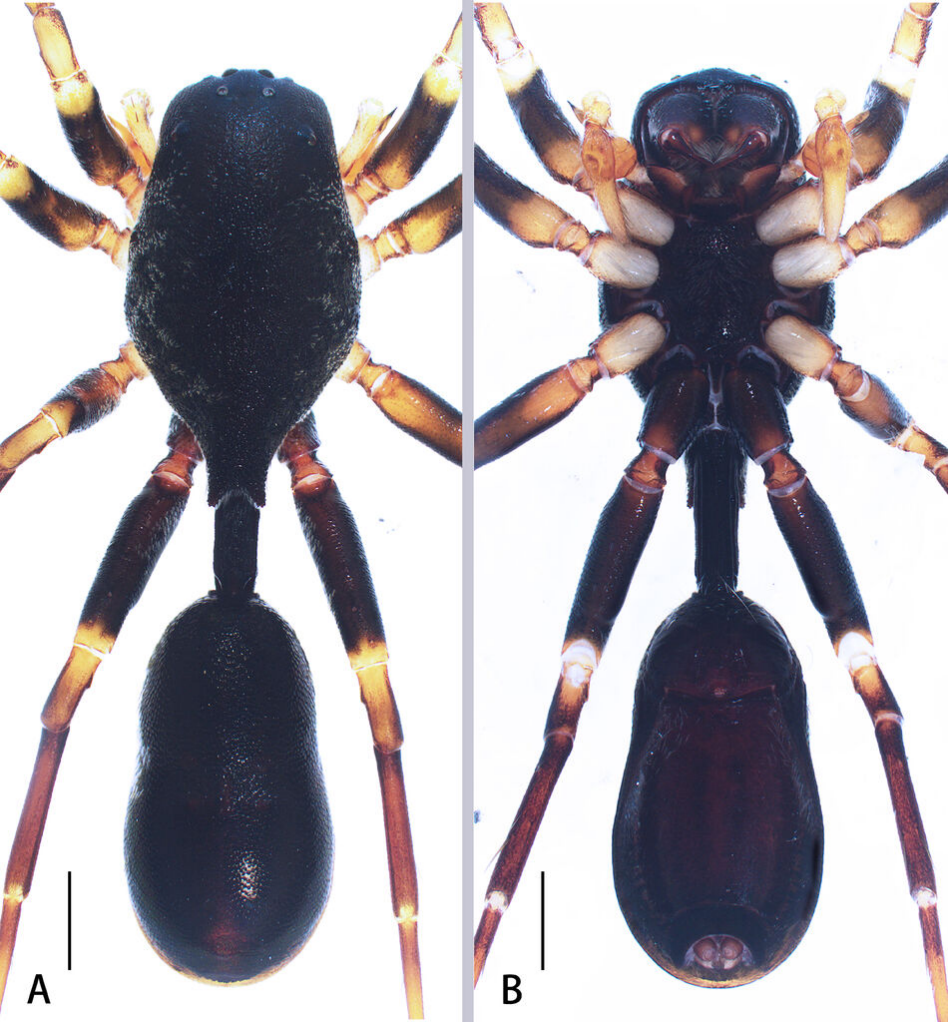
*Aetiusbicuspidatus* Yamasaki, 2020. **A** male habitus, dorsal view; **B** same, ventral view. Scales = 0.5 mm (A & B).

**Figure 5.**
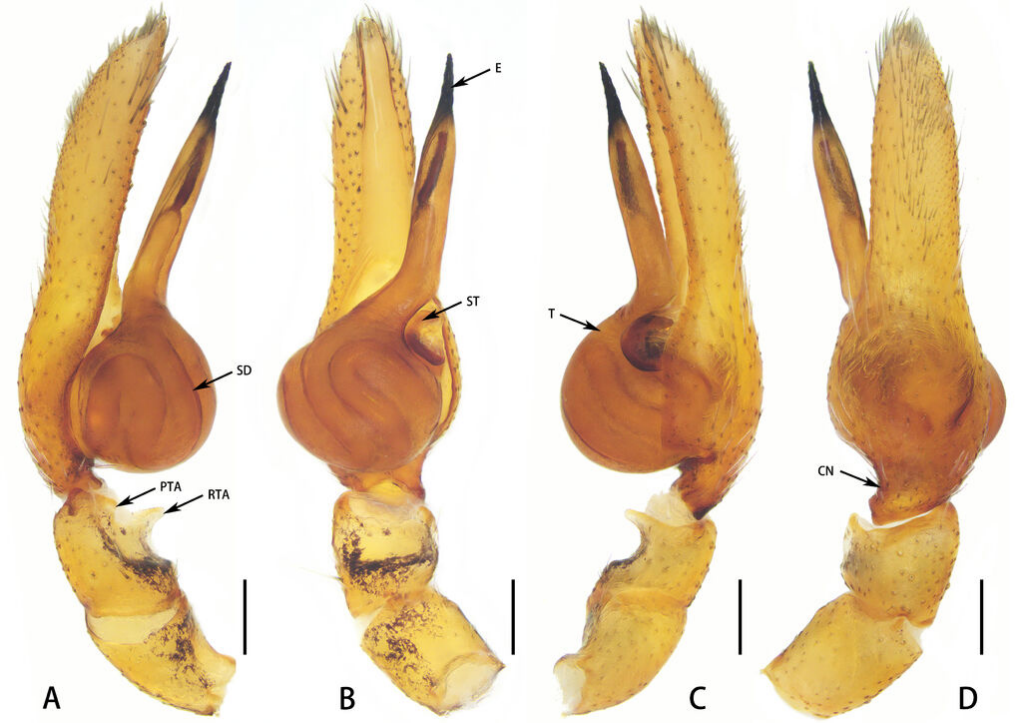
*Aetiusbicuspidatus* Yamasaki, 2020. **A** male left palp, prolateral view; **B** same, ventral view; **C** same, retrolateral view; **D** same, dorsal view. Abbreviations: CN—cymbial semicircular notch; E—embolus; PTA—prolateral tibial apophysis; RTA—retrolateral tibial apophysis; SD—sperm duct; ST—subtegulum; T—tegulum. Scales = 0.1 mm (**A–D**).

*Aetiusmaculatus* **sp. n.** can be distinguished from *A.bicuspidatus* by: 1) the trapezoidal posterior projection on the carapace (vs. the bicuspid posterior projection in *A.bicuspidatus*) (compare Fig. 2A and D with Fig. 4A); 2) ocular region with white setae (vs. absent in *A.bicuspidatus*) (compare Fig. 2C with Fig. 4A). Males can be further distinguished from those of *A.bicuspidatus* by: 1) the larger RTA, with weakly sclerotised edge; 2) the rounder and larger PTA; 3) the slender embolus (compare Fig. 3A–D with Fig. 5A–D). Females can be further distinguished by the short copulatory duct (vs. long in *A.bicuspidatus*) (compare Fig. 3E and F with figs. 2C and D in Yamasaki et al. 2020).

#### Etymology

The specific name is an adjective referring to the macule on the abdomen. Latin *maculatus* = macula.

#### Distribution

China (Yunnan Province) (Fig. 10).

### Aetius bicuspidatus

Yamasaki, 2020

9380E06A-6B94-549A-9E1F-B643F6F2D4CE

#### Materials

- **Type status:** Other material. **Occurrence:** recordedBy: Mengjiao Xu; individualCount: 2; sex: male; lifeStage: adult; occurrenceID: A982BFC5-A5A8-5D37-807E-635928FAA504; **Location:** country: China; stateProvince: Yunnan Province; county: Jinghong; locality: Wild Elephant Valley; verbatimElevation: 814; verbatimLatitude: 22°10′25.05″N; verbatimLongitude: 100°51′19.07″E; **Event:** year: 2022; month: 6; day: 5; **Record Level:** institutionID: the Museum of Hebei University; institutionCode: MBHU

#### Diagnosis

*Aetiusbicuspidatus* can be distinguished from all *Aetius* species by: 1) the bicuspid posterior projection on the carapace; 2) the long pedicel (Fig. 4). Males can be further distinguished from those of *A.maculatus* **sp. n.** by: 1) the triangular PTA (vs. rounder in *A.maculatus* **sp. n.**); 2) the thick embolus, with blunt top (vs. slender embolus, with sharp top in *A.maculatus* **sp. n.**) (compare Fig. 5 with Fig. 3A–D). Females can be further distinguished from those of *A.nocturnus* by the long and thin copulatory duct (compare fig. 2D in Yamasaki et al. 2020 with Fig. 8F).

##### Redescription

Male (Fig. 4). Total length 4.70; carapace 3.05 long, 1.65 wide; abdomen 2.82 long, 1.46 wide. Eye sizes and interdistances: AME 1.23, ALE 0.67, PME 0.80, PLE 0.70; AME–AME 0.27, AME–ALE 0.17, ALE–ALE 0.56, PME–PME 0.32, PME–PLE 0.47, PLE–PLE 0.98, ALE–PLE 0.49; MOA 0.17 long; anterior width 0.37, posterior width 0.42. Clypeal height 0.22. Chelicerae with two pro- (proximal smallest, distal largest) and two retromarginal teeth (same size). Labium 0.12 long, 0.36 wide; sternum 0.95 long, 0.80 wide. Measurements of legs: I 3.93 (1.15, 0.46, 0.87, 0.77, 0.68), II 3.97 (1.21, 0.45, 0.87, 0.78, 0.66), III 3.90 (1.21, 0.42, 0.90, 0.76, 0.61), IV 5.25 (1.50, 0.54, 1.24, 1.26, 0.71).

Carapace black, sub-pentagonal; carapace narrowed posteriorly, forming bicuspid; pedicel elongated (Fig. 4A). Legs Ⅰ-Ⅲ yellow, middle of femora black; Leg Ⅳ brown, distal femora, patellae and middle of metatarsi yellow. Abdomen black, oval, with medial constriction dorsally. Dorsal scutum large, nearly covered abdomen (Fig. 4B).

Palp (Fig. 5). Tibia concaved in ventral surface; retrolateral tibial apophysis digitiform, weakly sclerotised, slightly transparent; prolateral tibial apophysis triangular. Embolus conical, thick, with blunt top.

#### Distribution

Malaysia (Borneo), China (Yunnan Province) (Fig. 10).

### Aetius nocturnus

Deeleman-Reinhold, 2001

003DF1C2-0336-5DF5-A5A7-4D45DC8C90BB

#### Materials

- **Type status:** Other material. **Occurrence:** recordedBy: Chi Jin; individualCount: 3; sex: male; lifeStage: adult; occurrenceID: 89B674FC-757C-5F08-8635-46AD30DAFF3F; **Location:** country: China; stateProvince: Yunnan Province; county: Menghai; locality: Xishuangbanna Daluo Forest Park; verbatimElevation: 592; verbatimLatitude: 21°41′43.30″N; verbatimLongitude: 100°2′37.58″E; **Event:** year: 2018; month: 7; day: 28; **Record Level:** institutionID: the Museum of Hebei University; institutionCode: MHBU
- **Type status:** Other material. **Occurrence:** recordedBy: Yulong Wang; individualCount: 1; sex: male; lifeStage: adult; occurrenceID: 3A0730A0-B726-5184-A24A-DE299129CD2E; **Location:** country: China; stateProvince: Yunnan Province; county: Jinghong; locality: Mengyang Town; verbatimElevation: 600; verbatimLatitude: 22°4′33.97″N; verbatimLongitude: 100°52′14.57″E; **Event:** year: 2006; month: 7; day: 28; **Record Level:** institutionID: the Museum of Hebei University; institutionCode: MHBU
- **Type status:** Other material. **Occurrence:** recordedBy: Lu Zhan; individualCount: 1; sex: female; lifeStage: adult; occurrenceID: 1CB43EC3-A421-5163-9364-35685B11BE49; **Location:** country: China; stateProvince: Yunnan Province; county: Jinghong; locality: Wild Elephant Valley; verbatimElevation: 800; verbatimLatitude: 22°10′24.12″N; verbatimLongitude: 100°51′33.75″E; **Event:** year: 2021; month: 8; day: 4; **Record Level:** institutionID: the Museum of Hebei University; institutionCode: MHBU

#### Diagnosis

*Aetiusnocturnus* can be distinguished by the following characters: 1) the stalk-like posterior projection on the carapace (vs. bicuspid in *A.bicuspidatus* or trapezoidal in *A.maculatus* **sp. n.**) (compare Fig. 7A and C with Fig. 4A or Fig. 2A and D); 2) carapace covered with numerous hairs, forming radiating striae (vs. without radiating striae in *A.bicuspidatus* or sparse plumose hair, forming faint radiating striae in *A.maculatus* **sp. n.**) (compare Fig. 7A and C with Fig. 4A or Fig. 2A, C and D); 3) the slender embolus (vs. thick embolus in *A.bicuspidatus*) (compare Fig. 9C with Fig. 9B); 4) copulatory duct thick (vs. copulatory duct thin in *A.bicuspidatus*) (compare Fig. 8E and F with figs. 2C–D in Yamasaki et al. 2020); 5) posterior spermatheca wide (vs. posterior spermatheca narrow in *A.bicuspidatus*) (compare Fig. 8F with fig. 2D in Yamasaki et al. 2020).

##### Redescription

Male (Fig. 7A–B). Total length 6.74; carapace 4.17 long, 2.56 wide; abdomen 4.30 long, 2.44 wide. Eye sizes and interdistances: AME 0.18, ALE 0.13, PME 0.12, PLE 0.08; AME–AME 0.38, AME–ALE 0.22, ALE–ALE 0.77, PME–PME 0.56, PME–PLE 0.65, PLE–PLE 1.54, ALE–PLE 0.70; MOA 0.29 long; anterior width 0.46, posterior width 0.65. Clypeal height 0.43. Chelicerae with two pro- (proximal smallest, distal largest) and two retromarginal teeth (same size). Labium 0.26 long, 0.45 wide; sternum 1.47 long, 1.22 wide. Measurements of legs: I 7.65 (2.01, 0.68, 1.89, 1.68, 1.21), II 7.68 (2.14, 0.70, 1.97, 1.66, 1.21), III 7.36 (1.95, 0.80, 1.96, 1.63, 1.02), IV 9.29 (2.53, 0.91, 2.57, 2.17, 1.11).

Carapace black, sub-pentagonal, surface with numerous plumose hairs, forming radiating striae, widest at coxae II and III; carapace narrowed posteriorly, forming the stalk-like posterior projection on the carapace. Leg orange-red, retrolateral femora Ⅰ-Ⅱ and femora Ⅲ-Ⅳ black. Abdomen black, oval, with medial constriction dorsally; surface with short plumose hairs and a tuft of long white hair posteriorly. Dorsal scutum large, nearly covered abdomen (Fig. 7A–B).

Palp (Fig. 8A–D). Tibia remarkably concaved in ventral surface; retrolateral tibial apophysis digitiform, weakly sclerotised, curved ventrally; prolateral tibial apophysis arc-shaped, weakly sclerotised. Embolus conical, with screw thread.

**Figure 6.**
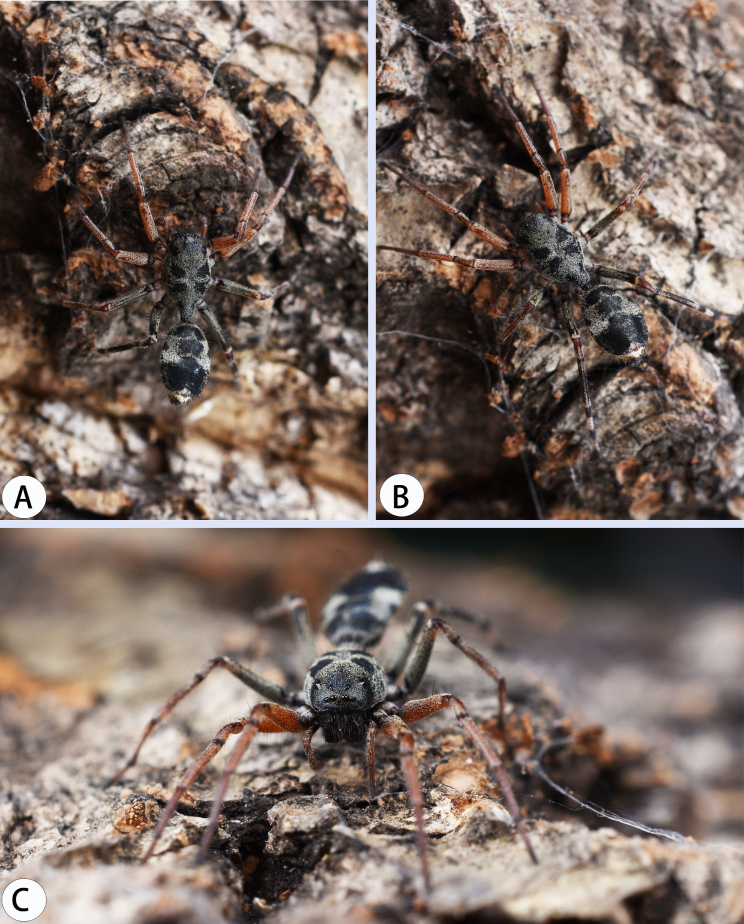
Living habitus of *A.nocturnus* Deeleman-Reinhold, 2001: **A–C** female (photographs by Weihang Wang).

**Figure 7.**
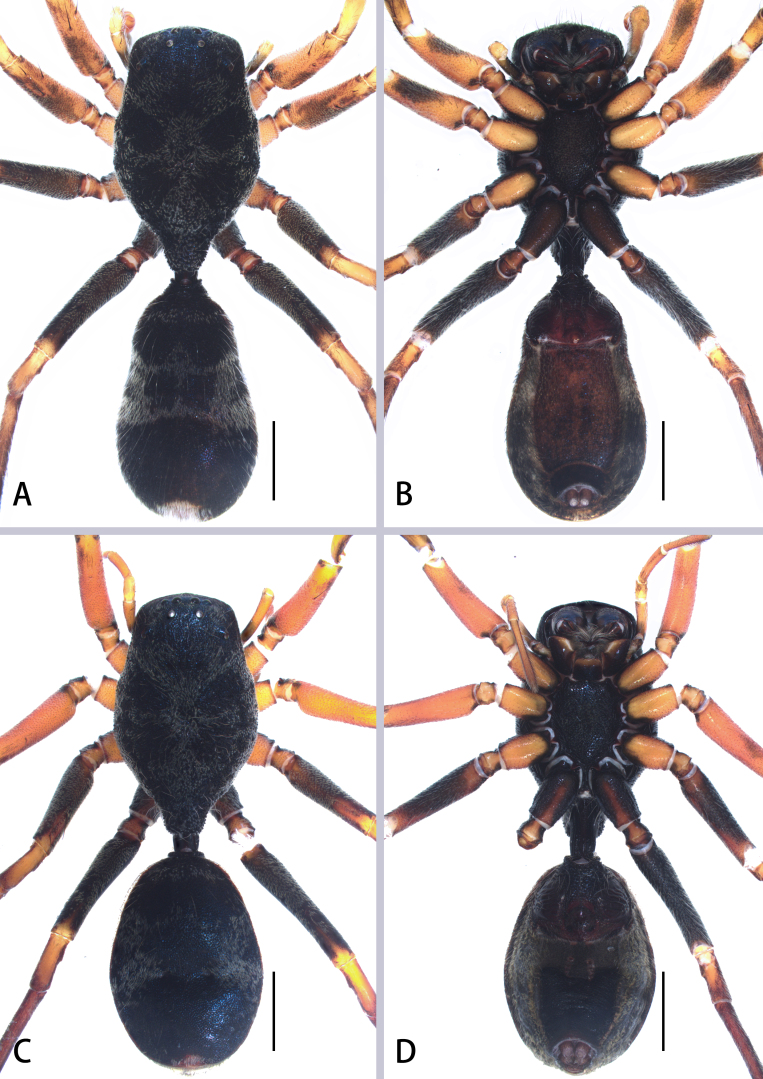
*Aetiusnocturnus* Deeleman-Reinhold, 2001. **A** male habitus, dorsal view; **B** same, ventral view; **C** female habitus, dorsal view; **D** same, ventral view. Scales = 0.5 mm (A–D).

Female (Figs 6, 7C–D). Total length 8.38; carapace 4.52 long, 2.70 wide; abdomen 3.86 long, 3.97 wide. Eye sizes and interdistances: AME 0.14, ALE 0.12, PME 0.11, PLE 0.10; AME–AME 0.37, AME–ALE 0.24, ALE–ALE 0.80, PME–PME 0.56, PME–PLE 0.48, PLE–PLE 1.56, ALE–PLE 0.55; MOA 0.33 long; anterior width 0.53, posterior width 0.67. Clypeal height 0.40. Labium 0.33 long, 0.57 wide; sternum 1.62 long, 1.22 wide. Measurements of legs: I 8.33 (2.49, 0.70, 2.08, 1.81, 1.25), II 8.40 (2.50, 0.72, 2.15, 1.78, 1.25), III 8.25 (2.47, 0.83, 2.15, 1.77, 1.03), IV 10.1 (2.96, 0.89, 2.74, 2.30, 1.21).

As in male, except abdomen, nearly round and leg orange-red, femora Ⅲ-Ⅳ black (Fig. 7C–D).

**Figure 8.**
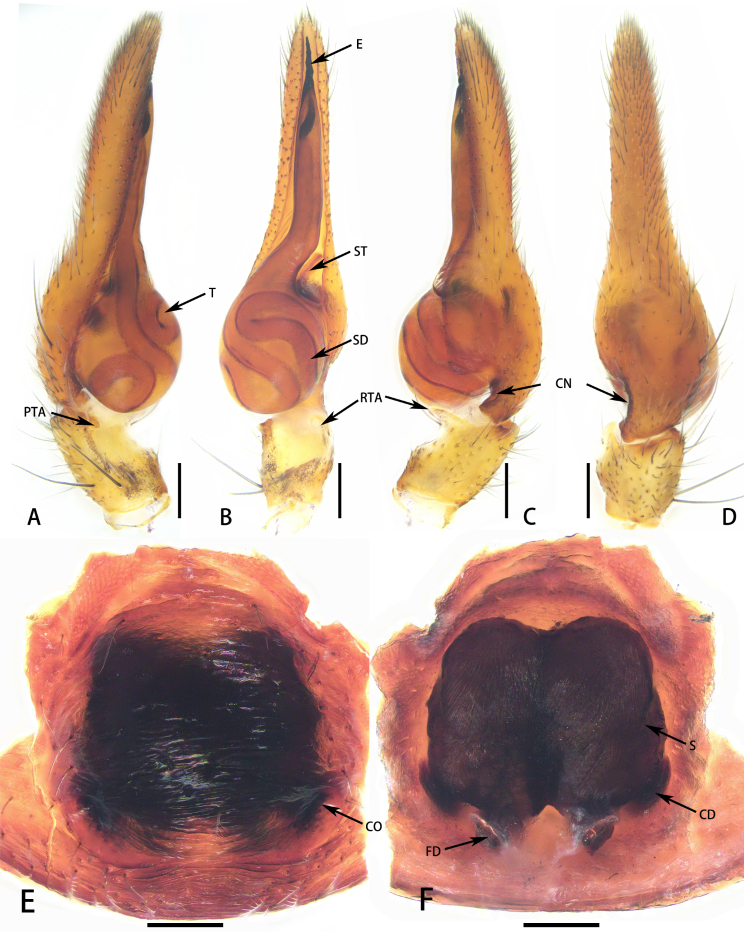
*Aetiusnocturnus* Deeleman-Reinhold, 2001. **A** Male left palp, prolateral view; **B** Same, ventral view; **C** Same, retrolateral view; **D** Same, dorsal view; **E** epigyne, ventral view; **F** same, dorsal view. Abbreviations: CD—copulatory duct; CO—copulatory opening; CN—cymbial semicircular notch; E—embolus; FD—fertilisation duct; PTA—prolateral tibial apophysis; RTA—retrolateral tibial apophysis; S—spermatheca; SD—sperm duct; ST—subtegulum; T—tegulum. Scales = 0.1 mm (A–F).

Epigyne characteristics as follow (Fig. 8E and F): simple, strongly sclerotised. Copulatory openings situated laterally in posterior of epigastric plate. Copulatory duct thick, connecting to spermatheca. Posterior spermatheca wide.

#### Distribution

Thailand, Malaysia (Borneo), China (Yunnan Province) (Fig. 10).

## Discussion

**Figure 9.**
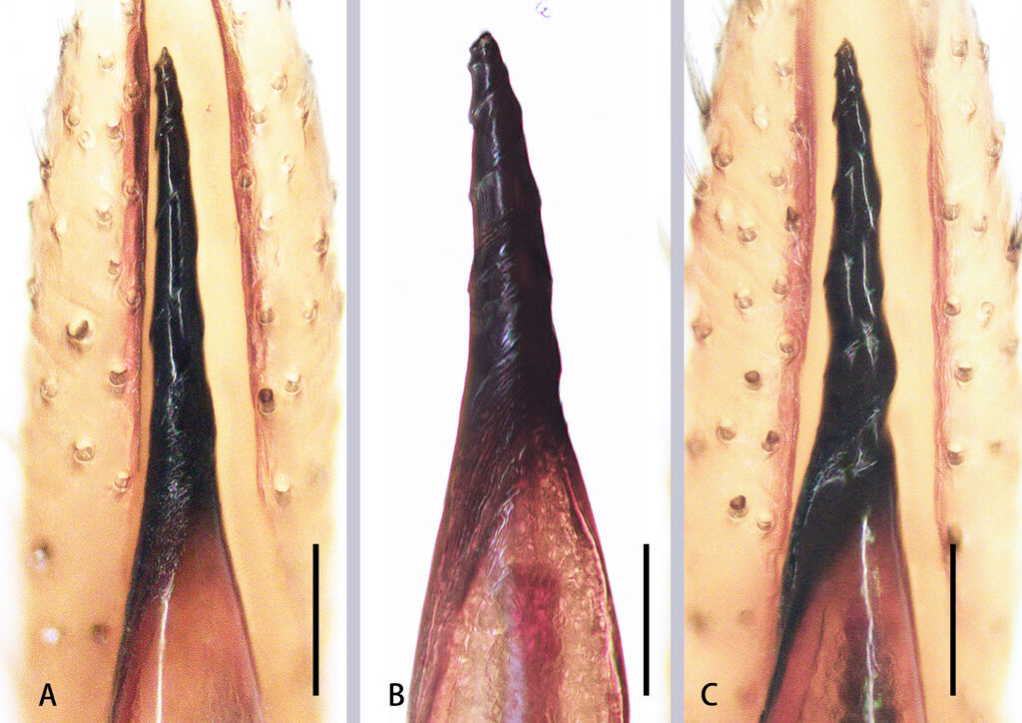
Emboli of three *Aetius* species. **A** *A.maculatus* sp. n.; **B** *A.bicuspidatus* Yamasaki, 2020; **C** *A.nocturnus* Deeleman-Reinhold, 2001. Scales = 0.1 mm (**A–C**).

**Figure 10.**
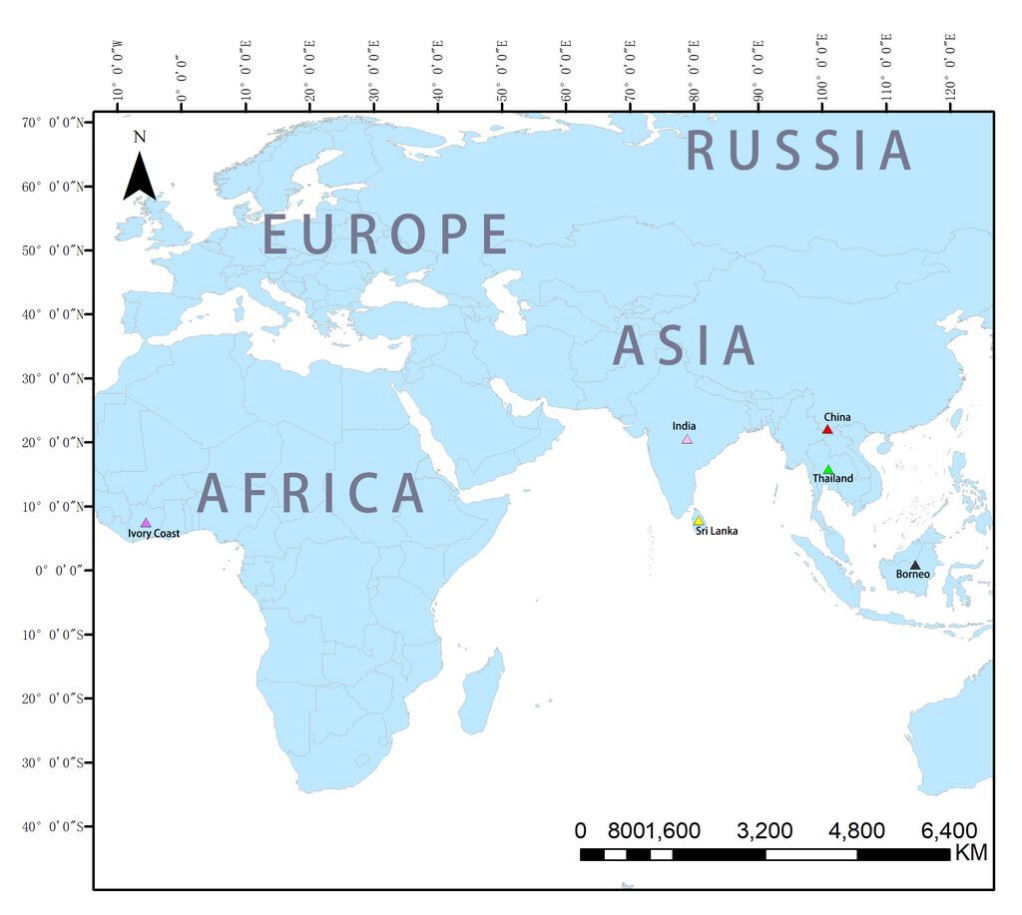
Distribution map of the genus *Aetius*.

Comparing the emboli of the three species, we found that some features are different (Fig. 9). Those which are different include: embolus slender, corkscrew-like and with sharp top in *A.maculatus* **sp. n.** (Fig. 9A); embolus thick, corkscrew-like and with blunt top in *A.bicuspidatus* (Fig. 9B); embolus slender, with distinct screw-thread whose terminal is close to the narrow part anteriorly of the bulb in *A.nocturnus* (Fig. 9C).

### Natural history

**Figure 11.**
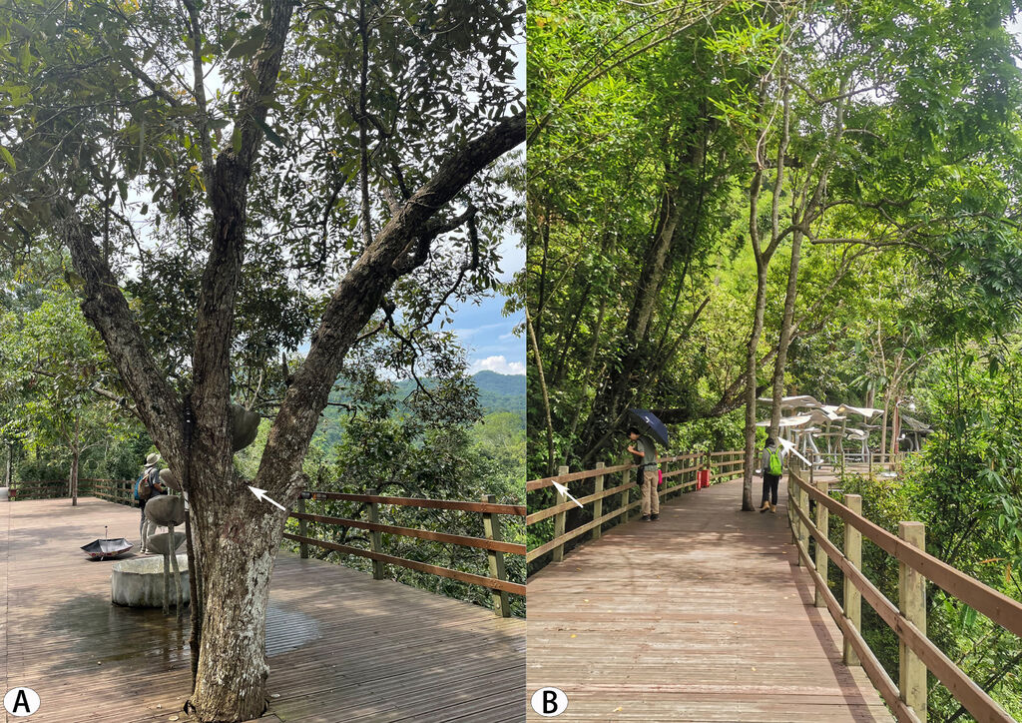
View of the collection sites of *Aetius* species: **A, B** Wild Elephant Valley (photographs by Lu Zhang and Mengjiao Xu).

The specimens were collected from Wild Elephant Valley, which is a tropical rainforest with little human damage. Three species were found near the tree canopy 2–5 metres above the ground. Meanwhile, we found that *Aetius* moved with the worker ants on tree trunks or railings of the skywalk (Fig. 11).

## Supplementary Material

XML Treatment for Aetius

XML Treatment for Aetius maculatus

XML Treatment for Aetius bicuspidatus

XML Treatment for Aetius nocturnus
